# DNMT/TET Imbalance and Network-Level DNA Methylation Remodeling in Ovarian Aging: Mechanistic Perspectives

**DOI:** 10.3390/biology15070577

**Published:** 2026-04-03

**Authors:** Miaofang Lin, Sheng Yang, Fengwen Huang, Xiaoyifan Deng, Chengwan Shen, Xiangkai Zhen, Aikebaier Reheman

**Affiliations:** 1 Fujian Key Laboratory of Toxicant and Drug Toxicology, Medical College, Ningde Normal University, Ningde 352100, China; linmiaofang@ndnu.edu.cn (M.L.); yangsheng@ndnu.edu.cn (S.Y.); 17759796371@163.com (X.D.); shchwan@sina.com (C.S.); 2 Department of Ophthalmology, Stanford University, Palo Alto, CA 94303, USA; fwhuang2@stanford.edu; 3 College of Life Sciences, Fujian Normal University, Fuzhou 350117, China

**Keywords:** reproductive aging, DNA methylation, DNMT–TET disequilibrium, network-level regulatory instability, follicular microenvironment, epigenetic biomarkers

## Abstract

Reproductive aging is characterized by progressive decline in ovarian reserve, reduced oocyte competence, and impaired endocrine coordination. Although these phenotypic changes are well documented, the molecular mechanisms that integrate aging-associated stress signals into coordinated ovarian dysfunction remain incompletely understood. Increasing evidence indicates that DNA methylation remodeling is closely associated with ovarian aging. Rather than representing isolated promoter-specific events, age-related methylation alterations may reflect progressive imbalance between DNA methyltransferases (DNMTs) and TET-mediated demethylation. Stress-responsive DNMT/TET dysregulation has been linked to distributed epigenetic remodeling across regulatory elements governing PI3K–AKT, TGF-β/SMAD, metabolic, and DNA damage response pathways in ovarian cell populations. We propose a network-level framework in which methylation drift preferentially affects highly connected regulatory hubs, potentially reducing transcriptional robustness and intercellular coordination within the follicular microenvironment. However, current human data remain largely correlative, and functional validation is required to determine whether methylation remodeling acts as a driver, amplifier, or biomarker of ovarian aging. Finally, we discuss translational implications, including circulating cell-free DNA signatures and epigenetic clock models, while emphasizing the importance of cell type-resolved and longitudinal studies. Collectively, the available evidence supports a model in which progressive DNMT/TET imbalance is associated with distributed pathway-level regulatory instability during ovarian aging.

## Simple Summary

Reproductive aging is marked by a gradual decline in ovarian reserve and oocyte quality, ultimately leading to reduced fertility. While hormonal changes have long been recognized, increasing evidence suggests that epigenetic regulation—particularly DNA methylation—may play an important integrative role in this process. Rather than reflecting isolated gene-specific changes, age-associated methylation alterations appear to involve the broader remodeling of interconnected signaling pathways that regulate follicle activation, metabolic balance, and genome stability. This review proposes that progressive imbalance between DNA methyltransferases (DNMTs) and TET demethylases may contribute to distributed regulatory instability across ovarian cell types. We summarize current evidence linking stress-related signaling to methylation remodeling and discuss the emerging potential of methylation-based biomarkers for assessing ovarian aging. At the same time, we highlight the need for cell type-resolved and longitudinal studies to clarify causal relationships. Although the field has made rapid progress, many mechanistic links remain unresolved, underscoring the need for cautious interpretation.

## 1. Introduction

Reproductive aging represents a progressive systems-level decline characterized by depletion of the follicular reserve, deterioration of oocyte competence, and impaired endocrine coordination [1,2]. Although these clinical features are well recognized, the molecular mechanisms that integrate diverse stress inputs into coordinated ovarian dysfunction remain incompletely defined [3,4]. Multiple epigenetic regulatory layers—including DNA methylation [5], histone modifications [6], non-coding RNA pathways [7], and more broadly, chromatin-level remodeling mechanisms [6]—have been implicated in reproductive aging.

**Figure 1:**
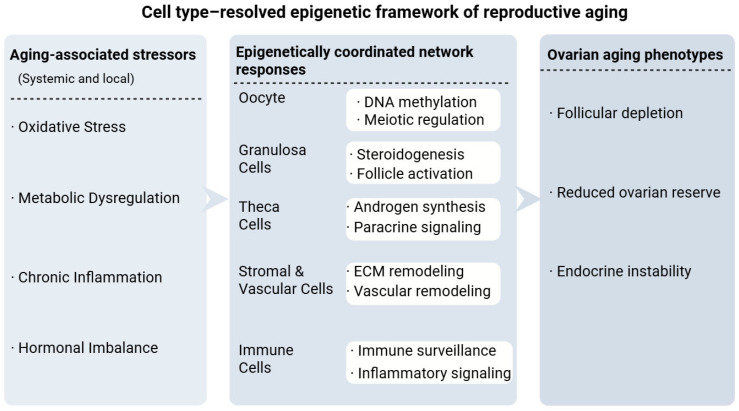
Cell type-resolved epigenetic integration of aging stressors and ovarian phenotypes. Conceptual model illustrating how aging-associated stressors converge on ovarian cell populations and reshape regulatory architecture through DNA methylation dynamics. Oxidative, metabolic, inflammatory, and endocrine signals interact with DNMT/TET-dependent methylation turnover, introducing asymmetry into regulatory element fidelity across oocytes, granulosa cells, theca cells, stromal/vascular compartments, and immune populations. Distributed epigenetic remodeling at hub-centered signaling pathways may reduce transcriptional robustness and intercellular coordination, thereby contributing to follicular depletion, diminished ovarian reserve, and endocrine instability.

To better understand this integrative process, reproductive aging can be conceptualized as a multilevel cascade linking systemic stressors to epigenetic remodeling and functional decline. Reproductive aging occurs within a multicellular ovarian environment composed of oocytes, granulosa cells, theca cells, stromal, vascular, and immune populations. Within this context, the aging process can be conceptualized as a three-layer framework linking systemic and local stressors to cell type-specific epigenetic responses and ultimately to overt ovarian phenotypes (Figure 1). Aging-associated stressors—including oxidative stress [8], metabolic dysregulation [9], chronic inflammation [10], and hormonal imbalance—do not act on a single ovarian compartment. Instead, they converge on a multicellular network composed of oocytes, granulosa cells, theca cells, and stromal, vascular, and immune populations [3,11].

Within this network, each cell type maintains a distinct epigenetic landscape that stabilizes lineage-specific transcriptional programs [11]. Oocytes coordinate meiotic regulation and genome integrity; granulosa cells regulate steroidogenesis and follicle activation; theca cells contribute androgen synthesis and paracrine signaling; stromal and vascular compartments modulate extracellular matrix and blood supply; and immune cells participate in inflammatory surveillance. Figure 1 integrates these cell type-resolved regulatory layers and highlights how stress-responsive epigenetic remodeling may differentially affect each compartment [12,13].

Among epigenetic mechanisms, DNA methylation has emerged as a relatively stable and integrative regulatory layer capable of embedding cumulative biological stress into chromatin architecture. However, most existing studies remain CpG-centric and descriptive. What remains insufficiently explained is why methylation-associated changes repeatedly converge on certain regulatory pathways during ovarian aging rather than appearing as fully random locus-specific events.

**Figure 2:**
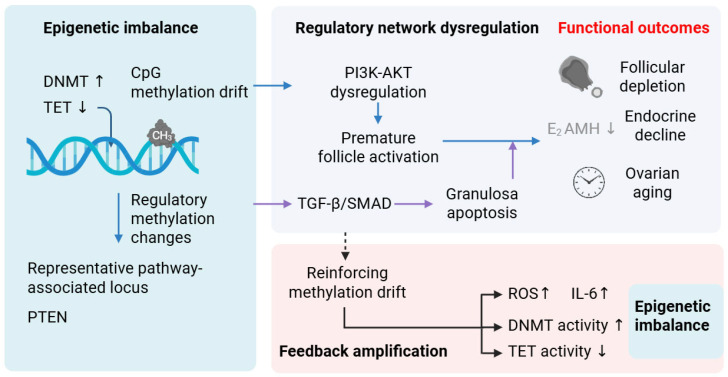
DNMT/TET imbalance may promote CpG methylation drift and regulatory methylation changes, potentially altering pathway responsiveness and functional coordination across ovarian cell populations. These DNMT/TET imbalance-associated methylation changes may be associated with disrupted signaling relevant to follicle activation, granulosa cell homeostasis, and broader ovarian functional decline. The convergence of methylation remodeling on PI3K–AKT and TGF-β/SMAD signaling is discussed in Section 2.3, with *PTEN* considered as a representative regulatory node in this framework. Stress-related factors such as reactive oxygen species (ROS) and inflammatory mediators may further reinforce methylation drift through feedback amplification. The model is intended to represent a network-oriented framework rather than deterministic gene-specific silencing.

Increasing evidence suggests that reproductive aging is accompanied by progressive imbalance in the dynamic turnover between DNA methyltransferases (DNMTs) and TET-mediated demethylation [5]. This imbalance does not reflect uniform global shifts. Instead, it may arise from altered metabolic substrate availability and oxidative modification of catalytic cofactors. Inflammation-associated transcriptional reprogramming may further reinforce this asymmetry [14]. Over time, asymmetry in methylation maintenance may contribute to the dysregulation of functionally interconnected signaling pathways [15]. These include representative pathways involved in follicle activation, granulosa cell homeostasis, metabolic regulation, and genome maintenance, such as PI3K–AKT–FOXO3 [16], TGF-β/SMAD [17], mTOR/AMPK [18], and DNA-damage [19] response pathways. Together, these networks coordinate follicle fate decisions across ovarian cell populations (Figure 2) [20,21]. Hence, we propose that DNMT/TET imbalance may contribute to the dysregulation of these pathways. This may occur through altered CpG methylation in the promoter regions and broader gene regulatory elements of key regulatory genes [14]. Accordingly, such distributed epigenetic perturbations could increase transcriptional variability and reduce the robustness of these hub-centered signaling networks. However, direct experimental evidence demonstrating selective pathway-level methylation remodeling driven by DNMT/TET imbalance in aging ovaries remains limited.

In this context, Figure 2 provides a conceptual scaffold illustrating how DNMT/TET imbalance may propagate across regulatory levels. At the molecular level, locus-specific methylation asymmetry may arise from altered DNMT/TET balance. At the pathway level, such asymmetry may destabilize hub-centered signaling modules. At the tissue level, disrupted intercellular coordination may ultimately contribute to ovarian functional decline [22,23]. This perspective shifts attention from isolated CpG alterations to coordinated pathway-level vulnerability. In contrast to previous reviews that primarily catalog locus-specific methylation changes [5,24,25,26,27,28], this review proposes a working framework in which DNMT/TET imbalance may preferentially affect highly connected signaling pathways. Within this interpretation, ovarian aging is viewed less as a deterministic promoter of hypermethylation and more as a progressive weakening of regulatory coordination associated with asymmetrical methylation turnover across regulatory elements.

## 2. DNA Methylation Regulation of Gene Expression in Ovarian Cell Types

In human ovarian granulosa cells, age-related DNA methylation changes are not randomly distributed but instead are associated with regulatory regions linked to gene expression remodeling and stress-responsive pathways [29]. Figure 2 summarizes our proposed framework, in which epigenetic imbalance may contribute to pathway dysregulation and, ultimately, to functional decline in the aging ovary. DNA methylation also exhibits marked cell type specificity across follicular and non-follicular compartments, consistent with the distinct regulatory states of ovarian cell populations [14].

In this review, the term “DNA methylation drift” refers to progressive, context-dependent deviations from homeostatic methylation states across promoters and distal regulatory elements, rather than uniform global hyper- or hypomethylation [30]. In ovarian aging, such drift appears to involve cell type-specific remodeling across follicular compartments, particularly in granulosa and cumulus cells, where methylation changes have been associated with altered transcriptional programs relevant to follicle activation, growth, and endocrine function [29,30,31]. Importantly, these changes are not confined to promoters but also involve distal regulatory elements and enhancer-associated CpG regions, thereby extending their potential effects beyond isolated gene-specific methylation changes (Figure 2) [32,33].

### 2.1. Genome-Wide Methylation Drift and Transcriptional Remodeling in Oocytes and Granulosa Cells

Genome-wide methylome analyses of human granulosa and cumulus cells have revealed widespread differentially methylated regions. Such changes were identified in women with diminished ovarian reserve or poor ovarian response to controlled ovarian stimulation during IVF [34]. In one of the more informative early studies, Yu et al. analyzed granulosa cells from older poor responders and younger good responders [29]. The study showed coordinated age-associated differences in both DNA methylation and transcriptomic profiles, although the methylation analysis was based on pooled samples and the overall cohort size remained modest. Poor ovarian response is typically assessed by a low number of retrieved oocytes together with abnormal ovarian reserve markers, including anti-Müllerian hormone and antral follicle count [35,36]. More recent studies in cumulus/granulosa cell populations have similarly reported transcriptomic and epigenetic alterations linked to impaired ovarian response, but most available data remain cross-sectional and derived from relatively small IVF-based cohorts [29,37]. Anti-Müllerian hormone (AMH) provides an illustrative example of granulosa cell functional alteration. Partial CpG methylation near the *AMH* locus has been associated with reduced *AMH* expression in granulosa cells from poor responders [29,38]. Because declining AMH levels are clinically associated with a reduced follicle number, the observed methylation-associated reduction in AMH expression may reflect impaired follicular support [39]. However, most human evidence remains cross-sectional and granulosa/cumulus-centered, limiting causal inference and broader cell type generalization [40,41]. Representative study characteristics are summarized in Table 1.

Methylation alterations have also been reported in diminished ovarian reserve and polycystic ovary syndrome, involving genes related to growth factor signaling, extracellular matrix remodeling, metabolic regulation, and inflammatory responses [29,42,43]. Although polycystic ovary syndrome is not a direct model of ovarian aging, these findings suggest that ovarian dysfunction in different contexts may involve partially overlapping methylation-sensitive regulatory pathways [44]. Multi-omics analyses indicate that DNA methylation changes occur in parallel with broader chromatin and transcriptional remodeling, particularly within PI3K–AKT, mTOR, and TGF-β signaling networks [45] (Figure 2). In oocytes, methylation shifts have been associated with the dysregulation of *ATM*- and *BRCA1*-dependent DNA damage repair pathways, potentially contributing to genome instability and reduced developmental competence [46,47,48]. Nevertheless, promoter-specific hypermethylation of individual DNA repair genes in aging human oocytes remains insufficiently characterized [49].

Parallel single-cell analysis of aged mouse oocytes further showed increased transcriptomic variability together with localized DNA methylation changes, supporting the view that aging-associated epigenetic perturbation may be spatially distributed rather than uniformly promoter-centered [44]. Together, these data support a model in which cumulative methylation remodeling reshapes transcriptional programs in follicular compartments. However, most human studies remain correlative, are based primarily on granulosa or cumulus cell material, and vary considerably in sample type, analytical platform, and locus selection. As summarized in Table 2, mechanistic support is currently stronger for selected loci and pathway contexts than for a consistent promoter-specific methylation signature across ovarian aging.

### 2.2. Convergence of Methylation Remodeling on PI3K–AKT and TGF-β/SMAD Signaling Networks

Age-associated methylation changes are distributed across many loci [29]. Their functional effects, however, appear to converge on a limited number of signaling networks that govern follicle fate decisions, particularly the PI3K–AKT and TGF-β–SMAD pathways [20]. Support for this view comes mainly from pathway enrichment analyses, integrative methylome–transcriptome studies, and coordinated transcriptional changes observed in granulosa and cumulus cells [62].

The PI3K–AKT pathway is a central regulator of primordial follicle activation, oocyte survival, and granulosa cell function [55]. In ovarian aging-related settings, methylation alterations have been associated with coordinated changes in genes linked to upstream regulators, feedback modulators, and downstream effectors within this pathway [26]. These findings suggest that epigenetic remodeling may reshape pathway responsiveness at the network level, even when key components such as *PTEN* do not exhibit uniform promoter-specific methylation [63]. Thus, the functional impact of methylation drift may arise less from silencing one master regulator than from cumulatively altering the balance, sensitivity, and signaling output of the broader PI3K–AKT regulatory network.

A distributed, pathway-level pattern is also evident for TGF-β–SMAD-related signaling, which is essential for oocyte–granulosa communication, follicular growth, and endocrine responsiveness [64,65,66]. Available evidence does not consistently support stable promoter methylation of canonical SMAD genes across ovarian aging contexts. Instead, current evidence suggests a broader pattern. Pathway enrichment analyses, integrative methylome–transcriptome studies, and coordinated transcriptional analyses in granulosa and cumulus cells all point to a consistent pattern. Methylation changes appear to be distributed across multiple regulatory loci and are accompanied by parallel pathway-level transcriptional alterations [67,68]. In this framework, age-related methylation remodeling may weaken the precision of communication between ovarian compartments rather than uniformly suppressing a single signaling axis [66].

Because most available evidence still derives from granulosa and cumulus cells, an important next step is to determine whether similar methylation remodeling also extends to stromal, vascular, and immune compartments.

### 2.3. Methylation Remodeling Beyond the Follicle

Beyond follicular and granulosa-centered regulatory changes, ovarian aging also involves the remodeling of the broader stromal, vascular, and immune microenvironment [69,70]. Current evidence suggests that this process is associated with stromal fibrosis and extracellular matrix remodeling, with extracellular matrix genes such as *COL1A1* reported to be upregulated in fibrotic ovarian stroma [70,71,72]. However, direct evidence connecting these non-follicular alterations to age-associated DNA methylation changes in stromal, vascular, or immune cell populations remains limited [69,70]. Studies of fibrotic remodeling in other tissues have shown epigenetic involvement in the regulation of extracellular matrix and inflammatory genes, raising the possibility that related mechanisms may also operate in ovarian stroma and immune populations [73]. At present, the lack of cell type-resolved methylome data from human ovarian stromal and immune compartments remains a major gap. This limitation underscores the need for single-cell and spatial epigenomic approaches to clarify how non-follicular epigenetic remodeling contributes to ovarian microenvironmental aging [74].

## 3. Stress-Responsive Drivers of DNMT/TET Imbalance in Ovarian Aging

Reproductive aging unfolds within a dynamic systemic milieu in which oxidative, metabolic, inflammatory, endocrine, and environmental signals continuously interface with the ovarian epigenome [75,76,77]. Among these influences, oxidative stress and metabolic dysregulation represent two of the most consistently implicated drivers of ovarian epigenetic instability. Rather than acting as isolated insults, these stressors converge on the regulatory balance between DNA methyltransferases (*DNMT1*, *DNMT3A*, *DNMT3B*) and TET-mediated demethylation, progressively reshaping methylation fidelity across ovarian cell populations [76].

**Figure 3:**
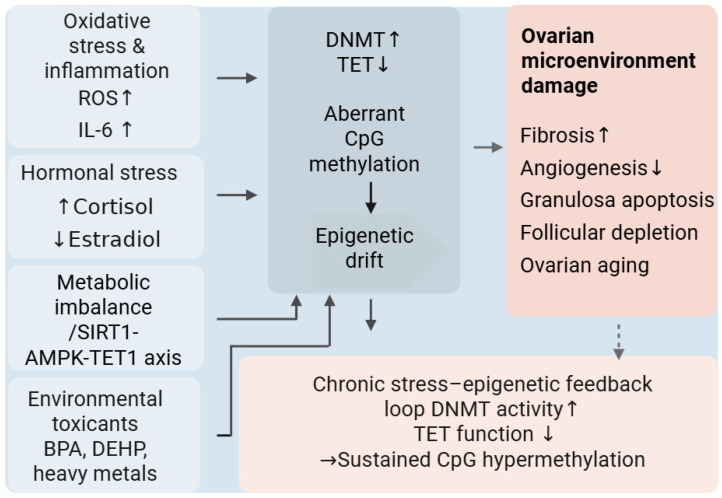
Schematic model of stress-induced DNMT/TET imbalance and ovarian epigenetic drift. Multiple intrinsic and extrinsic stressors, including oxidative and inflammatory cues, hormonal imbalance, metabolic dysfunction, and environmental toxicants, may contribute to DNMT/TET imbalance and epigenetic drift. DNMT/TET imbalance and epigenetic drift induced by these stressors may impair ovarian microenvironment integrity, promoting fibrosis, reduced angiogenesis, granulosa apoptosis, follicular loss, and ovarian aging. Chronic stress may interact with epigenetic remodeling in a self-reinforcing manner, potentially sustaining CpG hypermethylation and contributing to functional decline.

Importantly, stress-induced epigenetic remodeling does not necessarily present as uniform DNMT upregulation or global TET suppression. Instead, chronic stress introduces asymmetry into methylation maintenance cycles. This process may arise from altered substrate availability, redox imbalance, disrupted cofactor homeostasis, changes in chromatin accessibility, and inflammatory signaling cascades [78]. As illustrated in Figure 3, diverse intrinsic and extrinsic stressors may converge on DNMT/TET regulatory balance, thereby introducing asymmetry into methylation maintenance and promoting pathway-level destabilization.

### 3.1. Oxidative Stress as a Primary Driver of DNMT/TET Imbalance and PI3K–AKT–FOXO3 Destabilization

The ovary is particularly vulnerable to oxidative stress due to its high metabolic turnover and steroidogenic activity [2,79]. Reactive oxygen species can directly affect epigenetic enzyme activity, including DNMT recruitment and TET catalytic efficiency, thereby linking cellular redox imbalance to altered DNA methylation dynamics [80]. Accumulation of reactive oxygen species (ROS) during aging or toxicant exposure perturbs cellular redox balance and can directly influence the activity of epigenetic enzymes [81,82]. TET enzymes require α-ketoglutarate, Fe^2+^, and molecular oxygen as essential cofactors for catalytic demethylation [83,84]. Oxidative stress may disrupt these cofactors and impair TET function, favoring retention of methylated cytosines [78,85]. In parallel, inflammatory signaling, particularly NF-κB activation induced by cytokines such as IL-6 and TNF-α, has been associated with increased DNMT1 expression and altered chromatin recruitment [86]. Together, this stress-induced epigenetic imbalance may predispose ovarian cells to a hypermethylation-prone state [87,88], although ovary-specific, locus-level validation remains limited. Animal studies further demonstrate that oxidative insults induced by H_2_O_2_ [89] or environmental toxicants [90,91] alter methylation profiles associated with follicle development and early embryonic competence. Whether such changes represent stable epigenetic reprogramming or reversible stress responses in human ovarian tissues remains unresolved.

One possible consequence of oxidative stress-associated DNMT/TET imbalance is the altered regulation of the PI3K–AKT–FOXO3 axis, which plays a central role in maintaining primordial follicle quiescence [92]. The activation of primordial follicles is tightly constrained by inhibitory regulators such as *PTEN* and *FOXO3*, which maintain quiescence by restraining PI3K–AKT signaling [92]. Oxidative stress and accumulating DNA damage associated with aging intersect with epigenetic alterations, contributing to age-related changes in chromatin states [80]. Mechanistically, oxidative DNA damage can recruit DNA methyltransferases to damaged sites, directly linking ROS to alterations in methylation patterns [20]. More broadly, accumulated oxidative and metabolic stress modifies epigenetic landscapes over time and contributes to the progressive remodeling of regulatory hierarchies with age [93]. In a similar regulatory context, CpG redistribution within *PTEN*- or *FOXO3*-associated enhancers could plausibly reduce transcriptional responsiveness without inducing complete promoter silencing. Such regulatory perturbations may subtly bias PI3K–AKT pathway activity and lower the activation threshold of primordial follicles. This model suggests that DNMT/TET imbalance reshapes pathway dynamics through regulatory element instability rather than complete gene silencing [20]. Among the stress-related mechanisms discussed thus far, oxidative stress is currently supported by comparatively direct links to DNMT/TET imbalance, altered methylation turnover, and pathway-level regulatory disruption in ovarian aging contexts.

### 3.2. Metabolic Dysfunction, One-Carbon Flux, and Energetic Imbalance as Central Determinants of Methylation Homeostasis

DNA methylation is tightly coupled to cellular metabolism, in part because the universal methyl donor S-adenosylmethionine (SAM) supplied by one-carbon metabolism directly fuels DNMT-mediated methylation reactions [94]. Perturbations in metabolic pathways can therefore reshape methylation homeostasis by altering SAM availability and redox balance, providing a mechanistic link between metabolic stress and epigenetic remodeling in ovarian cells [94].

Biochemical studies show that perturbations in one-carbon flux can alter DNA methylation homeostasis through effects on the SAM:SAH ratio and DNMT activity [95,96,97]. One-carbon metabolites are generated through the methionine cycle and are essential for DNMT-dependent methylation, further linking nutrient and metabolic status with epigenetic regulation [98,99,100]. Clinical and experimental evidence indicates that obesity and insulin resistance are associated with genome-wide methylation changes in metabolically active tissues such as adipose tissue and skeletal muscle [101,102,103]. These findings support the broader concept that metabolic stress can reshape epigenetic regulation. In the ovarian context, altered DNA methylation patterns have also been reported in polycystic ovary syndrome, including in granulosa and granulosa–lutein cells [104,105], although direct evidence linking these metabolic disturbances to DNMT/TET dysregulation during ovarian aging remains limited. Metabolic dysfunction therefore represents a biologically plausible contributor to ovarian methylation instability, particularly through altered methyl donor availability and redox balance, although ovary-specific mechanistic evidence remains less direct than for oxidative stress.

### 3.3. Environmental, Endocrine, Nutritional, and Circadian Influences Requiring Further Investigation

Environmental toxicants, endocrine signaling changes, nutritional status, and circadian disruption may also influence ovarian epigenetic stability [60,61]. Endocrine signaling changes can reshape DNA methylation programs during follicular development [61]; nutritional status can influence TET-dependent DNA demethylation in female germ cells [62]; and circadian disruption has been associated with altered epigenetic markers and impaired oocyte quality [106]. Experimental studies have shown that endocrine-disrupting chemicals such as bisphenol A, phthalates, and certain heavy metals can alter DNA methylation patterns in ovarian and germline tissues. Such studies have reported methylation changes in steroidogenesis-related genes, imprinted loci, and other genes involved in reproductive epigenetic regulation [61]. Nutritional and circadian influences are also biologically plausible, particularly through effects on methyl donor availability, mitochondrial function, and oxidative stress [96,97]. More broadly, environmental stressors have been shown in multiple systems to promote mitochondrial reactive oxygen species generation and redox imbalance, but comparable ovary-specific evidence at the subcellular level remains limited [99]. Environmental and metabolic stressors may contribute to oxidative imbalance and epigenetic dysregulation in ovarian aging. However, the precise intracellular origin of these effects and their direct impact on DNMT/TET regulation in specific ovarian cell types remain insufficiently resolved [100].

## 4. Methylation-Driven Pathway Rewiring in Ovarian Aging

From a systems-level perspective, this section moves beyond pathway association to consider how age-associated DNA methylation drift may contribute to network fragility and regulatory rewiring in ovarian aging [20]. Rather than focusing on isolated methylation changes at individual loci, the emphasis here is on how cumulative epigenetic remodeling may disturb communication among interconnected pathways involved in genome maintenance, stress adaptation, and ovarian functional decline [105]. In this context, methylation drift is considered not simply as a locus-specific event, but as part of a broader process through which regulatory robustness may gradually erode across interacting ovarian cell populations [69].

Importantly, age-associated methylation drift may do more than simply accompany ovarian dysfunction. It may also contribute to the progressive reconfiguration of regulatory hierarchies, including PI3K-AKT, TGF-β/SMAD, mTOR, and DNA damage response networks [15,21,29,105]. Rather than causing uniform gene silencing, methylation remodeling may preferentially affect nodal regulators whose central positions within signaling networks allow broader downstream effects. For example, perturbation of PTEN-associated regulatory elements could influence PI3K–AKT pathway responsiveness, whereas disruption of *BRCA1*- and *ATM*-related genome maintenance networks may impair oocyte survival and accelerate ovarian functional decline [46,107,108,109].

### Coupling of Genome Maintenance Networks to Methylation Drift

Oocyte longevity depends on efficient *BRCA1*-mediated homologous recombination and *ATM*-dependent checkpoint activation [48]. Persistent oxidative stress and DNA damage can promote chromatin remodeling and local methylation-related changes at damage-associated and repair-related loci [110], consistent with the broader framework of stress-induced DNMT/TET imbalance shown in Figure 3. Reduced TET-mediated demethylation may further impair the dynamic epigenetic regulation required for efficient repair responses [111]. Human studies have suggested that *BRCA1* mutations may be associated with reduced ovarian reserve and poorer fertility preservation outcomes, although not all cohorts have shown consistent differences [112]. In mouse models, the conditional loss of *Brca1* in oocytes causes ovarian reserve depletion and impairs oocyte maturation with advancing reproductive age [57].

Together, the human and mouse evidence support a model in which impairment of genome maintenance pathways contributes to ovarian functional decline, while associated epigenetic dysregulation may further reinforce this process in a feed-forward manner [110,113]. In turn, compromised repair capacity activates CHK2-dependent DNA damage signaling, leading to the activation of p53- and TAp63-mediated apoptotic pathways and accelerating oocyte loss [114,115]. Although direct locus-specific methylation mapping in aging human oocytes remains limited, DNA damage signaling and methylation imbalance can still be integrated into a mechanistic framework. This proposed relationship is also consistent with the mechanistic framework summarized in Figure 3. Although current human evidence remains largely correlative, this framework provides a useful basis for future biomarker development and for causal testing of whether methylation remodeling in genome maintenance and stress response pathways directly contributes to oocyte loss and ovarian functional decline.

## 5. Epigenetic Biomarkers and Translational Perspectives

**Figure 4:**
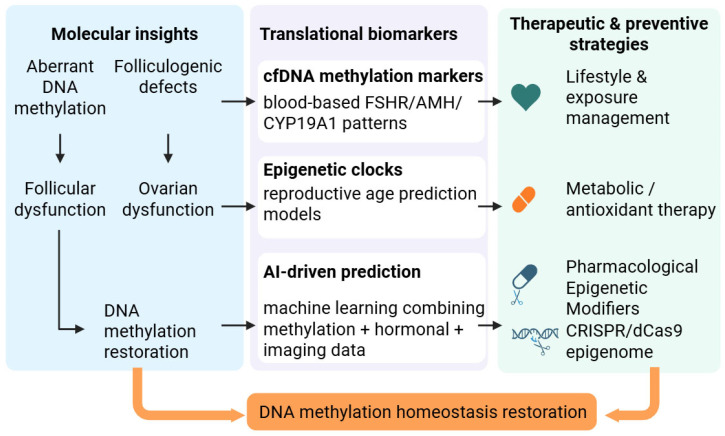
Translational framework linking ovarian DNA methylation drift to biomarker development and epigenetic intervention strategies. Molecular disturbances such as aberrant DNA methylation and folliculogenic defects contribute to follicular and ovarian dysfunction, motivating efforts to restore methylation homeostasis. In the translational domain, multiple biomarker modalities—including cfDNA methylation signatures, epigenetic clocks, and AI-driven predictive models—offer emerging tools for assessing reproductive aging. Such biomarker modalities also inform therapeutic and preventive strategies, ranging from lifestyle and exposure management to metabolic/antioxidant therapy, pharmacological epigenetic modifiers, and CRISPR/dCas9-based epigenome editing, collectively aiming to re-establish DNA methylation homeostasis. Antioxidant and other intervention strategies, including antioxidant approaches, are discussed further in Section 5.4 and remain conceptual pending further validation.

The identification of reliable biomarkers for ovarian aging remains a central challenge in reproductive medicine. Conventional clinical indicators—including serum FSH, AMH, and antral follicle count—often reflect relatively advanced functional decline and exhibit substantial inter-individual variability [116]. In contrast, DNA methylation may capture cumulative biological exposure and could provide earlier molecular insight into reproductive aging trajectories [41]. However, translating mechanistic epigenetic findings into clinically actionable biomarkers remains challenging and requires rigorous assessment of tissue specificity, longitudinal stability, technical reproducibility, and predictive performance. At present, most methylation-based observations in ovarian aging are derived from cross-sectional or correlative studies and therefore remain exploratory rather than validated diagnostic tools [117]. Figure 4 summarizes this translational trajectory, linking stress-induced methylation remodeling to emerging biomarker strategies and potential intervention frameworks.

### 5.1. Circulating cfDNA Methylation

Cell-free DNA (cfDNA) carries tissue-of-origin methylation signatures and offers a minimally invasive window into organ-specific epigenetic states [118]. Advances in bisulfite sequencing, targeted CpG assays, and fragmentomic analysis have markedly improved the sensitivity of cfDNA profiling [119]. In oncology, cfDNA methylation patterns have been used for cancer detection, classification, and tissue-of-origin inference [120,121]. Prenatal studies demonstrate that biologically informative tissue-derived DNA signals can be recovered from circulation [122,123]. Oncology and prenatal cfDNA studies establish the technical feasibility of methylation-based liquid biopsy approaches, but they do not constitute direct evidence for ovarian disease or ovarian aging biomarkers [122,124]. Rather, they provide a methodological foundation for investigating whether ovarian-derived methylation signals may eventually be detectable in circulation [123]. In the ovarian aging context, several observations suggest that methylation-associated abnormalities can be detected in association with early ovarian reserve decline. Cross-sectional studies in granulosa and related follicular somatic cells have reported DNA methylation abnormalities associated with diminished ovarian reserve and poor ovarian response, including changes involving ovarian function-related genes such as *AMH* [29,34]. More recent work has also shown that granulosa cells’ epigenetic age acceleration is associated with lower AMH, lower antral follicle count, and poorer ovarian response during IVF [40,125].

Together, these observations from granulosa/follicular somatic cell methylation studies and blood-based epigenetic age acceleration analyses provide a rationale for investigating whether ovarian dysfunction leaves detectable circulating epigenetic traces. As illustrated in the first translational branch of Figure 4, cfDNA methylation is being explored as a minimally invasive biomarker modality, although ovarian-specific applications remain unvalidated. Nevertheless, ovarian-specific cfDNA methylation panels have not yet been validated for predicting diminished ovarian reserve or premature ovarian insufficiency, and direct evidence linking ovarian methylation drift to detectable circulating cfDNA signals remains limited [118]. Critical challenges include the low fractional contribution of ovarian-derived cfDNA in peripheral blood, potential confounding by systemic aging signals, and the need for well-powered longitudinal cohorts [126,127]. Accordingly, while cfDNA methylation represents a promising direction, its application to reproductive aging remains investigational for ovarian reserve decline, DOR/POI risk assessment, and longitudinal monitoring [128].

### 5.2. Epigenetic Clocks and Reproductive Aging

Epigenetic clocks are typically constructed by identifying panels of age-informative CpG sites from DNA methylation datasets. Predictive algorithms are trained against chronological age or reproductive phenotypes [129,130]. The resulting models are subsequently evaluated by testing whether epigenetic age acceleration is associated with ovarian reserve markers, ART response, or reproductive lifespan-related outcomes. DNA methylation-based epigenetic clocks have emerged as robust estimators of biological age across multiple tissues [131].

Recent efforts to construct reproductive or ovarian-oriented clocks suggest that accelerated epigenetic aging may be relevant to ovarian function [132]. In women undergoing assisted reproduction, blood-based epigenetic age acceleration has been associated with decreased oocyte yield and poor ovarian response [36,133]. More recently, follicular fluid epigenetic age acceleration was also reported to correlate with IVF-related markers of ovarian response [108]. In parallel, population-level analyses have linked epigenetic clock variation to menopausal timing and broader reproductive lifespan characteristics [134,135]. However, current reproductive clocks are derived from relatively small cohorts and often rely on peripheral blood rather than ovarian tissue. External validation across diverse populations and longitudinal designs is still required. An additional unresolved question concerns causality [136]. Whether epigenetic age acceleration contributes directly to ovarian decline or instead reflects broader systemic aging remains unclear. Clarifying this distinction will be essential before epigenetic clocks can be implemented as predictive or stratification tools in clinical practice.

### 5.3. Artificial Intelligence and Multi-Modal Integration

The high dimensionality of DNA methylation datasets has stimulated machine-learning approaches for biomarker discovery, although small sample sizes and inadequate validation can lead to overfitting and limited clinical translation [137]. Supervised models such as SVM and random forests have been applied to cumulus cell methylation profiles to predict pregnancy outcomes after ICSI–IVF [138]. In oncology, machine-learning and deep-learning frameworks using cfDNA methylation—including models integrating methylation with fragmentomic features—have demonstrated high diagnostic or tissue-of-origin performance, illustrating the feasibility of methylation-based liquid biopsy modeling [139,140]. Nevertheless, most ovarian aging applications remain exploratory, with major limitations including technical heterogeneity/batch effects in methylation data, limited interpretability, and a lack of prospective validation. Future progress will likely require multi-modal integration of methylation with transcriptomic, metabolomic, hormonal, and imaging datasets to improve prediction and mitigate overfitting inherent to single-omic models [141]. However, as summarized in the second panel in Figure 4, we envisage that reproductive- or ovarian-oriented epigenetic clocks could be usefully investigated as future tools for biological age assessment in reproductive aging.

### 5.4. Clinical Translation and Therapeutic Considerations

Despite increasing interest, several challenges constrain the clinical translation of methylation-based biomarkers. Tissue specificity remains a central issue, as ovarian aging is a cell type-heterogeneous process revealed by recent single-cell and spatial atlas studies [142]. Technical variability in bisulfite-based profiling, sequencing depth, and computational normalization workflows further complicates cross-study comparability [143]. Moreover, ovarian aging unfolds over decades, necessitating well-powered longitudinal cohorts to establish predictive utility and clinical thresholds [99]. Ethical considerations also arise when contemplating epigenetic intervention strategies in reproductive contexts, including issues of risk, oversight, consent, and equity, and emerging debates specific to epigenome editing [144].

From a therapeutic perspective, conceptual approaches include lifestyle modification. They also include reduced exposure to environmental toxicants, endocrine-disrupting chemicals, metabolic stressors, and other adverse lifestyle-related factors [145]. At present, these interventions remain largely theoretical or preclinical in the context of ovarian aging, although NAD^+^ precursor supplementation and epigenome-editing toolkits provide proof-of-principle in animal and cellular systems [146]. Table 3 synthesizes representative methylation alterations reported in ovarian aging, alongside their current evidence status.

## 6. Conclusions

Overall, the evidence reviewed here is more consistent with a model of distributed regulatory remodeling across interacting ovarian cell populations than with one based solely on isolated gene-specific methylation lesions. This interpretation is supported by several observations discussed in the review. First, human granulosa and cumulus cell methylome studies have identified widespread methylation-associated changes linked to diminished ovarian reserve or poor ovarian response, although these data remain largely correlative [41], as discussed in Section 2.1 and summarized in Figure 2. Second, several canonical pathway components implicated in ovarian aging, including *PTEN* and canonical *SMAD* family members, do not consistently show promoter-specific methylation changes in human ovarian aging, arguing against a simple single-gene silencing model [17,34,149], as considered in Section 2.2 and integrated into the mechanistic framework in Figure 3. Third, emerging single-nuclei multi-omics evidence suggests that ovarian aging involves coordinated transcriptomic and epigenomic alterations across multiple ovarian cell populations rather than isolated defects within a single compartment, as discussed in Section Coupling of Genome Maintenance Networks to Methylation Drift and further summarized in Figure 3. Within this framework, epigenetic remodeling may help integrate cumulative stress exposure at the pathway level, potentially contributing to ovarian dysfunction during aging [62].

This perspective raises several important questions for future research. If core pathway genes are not consistently promoter-methylated, where do the primary regulatory perturbations occur? How do distributed methylation changes across distal regulatory elements, enhancer-associated regions, and pathway-linked control nodes reshape signaling responsiveness in specific ovarian cell populations? Addressing these questions will require cell type-resolved epigenomic profiling together with epigenome-editing approaches that are now enabling more direct testing of causal relationships [150]. Clarifying these mechanisms will be essential for understanding how epigenetic instability contributes to ovarian aging and for guiding the future development of biomarker and intervention strategies.

## Figures and Tables

**Table 1:** Representative primary studies and contextual reviews relevant to epigenetic and transcriptional alterations in ovarian dysfunction.

Study	Sample Source and Size/Scope	Key Finding and Limitation
Yu et al., 2015 [29]	Human ovarian granulosa cells from young oocyte donors and older poor responders; 20 vs. 20, with RNA-seq in 6 vs. 6 and methylation analyses in pooled sets of 10 vs. 10	Reported coordinated transcriptomic and methylomic differences in granulosa cells and highlighted *AMH* as an illustrative locus. Limited by cross-sectional design and partial reliance on pooled DNA.
Liu et al., 2022 [37]	Human cumulus granulosa cells from women with diminished ovarian reserve and normal ovarian reserve; 25 vs. 25	Identified broad transcriptomic alterations related to follicular function and signaling pathways. Informative for pathway disruption, but not direct evidence of DNA methylation change.
Wang et al., 2023 [38]	Literature-based review; no single cohort applicable	Summarized epigenetic mechanisms in premature ovarian failure/insufficiency, but does not provide primary cohort-level evidence.
Greene et al., 2014 [39]	Systematic review of published studies; 21 studies plus 2 case reports	Summarized genetic associations with diminished ovarian reserve, but provides indirect rather than direct evidence for methylation remodeling.

**Table 2:** Representative evidence supporting pathway-associated methylation remodeling in ovarian aging and related dysfunction.

Evidence Context	Representative Loci	Biological Relevance	Cell Type(s)/ Material	Key Implication
Human POI integrative [50,51]	*FSHR*, *NOBOX*, *GDF9*, *PTEN*; DNA repair loci	Folliculogenesis, ovarian reserve, genome integrity	Ovarian tissue, granulosa/cumulus cells, clinical samples	Supports distributed methylation-associated dysregulation in POI without consistent promoter-specific convergence on a single hub gene.
*GDF9* methylation (mechanistic) [52]	*GDF9* promoter CpG site	Oocyte signaling	Reporter and mechanistic models	Supports functional relevance of locus-specific methylation in oocyte-associated regulation.
*PTEN*–PI3K–AKT cross-talk with genome integrity [53,54,55]	*PTEN*, *PI3K*, *AKT*	Follicle activation, growth signaling, genome surveillance	Oocytes, ovarian tissue, animal models	Supports pathway-level vulnerability, while consistent *PTEN* promoter hypermethylation remains unconfirmed.
*BRCA*–oocyte genomic instability [56,57,58]	*BRCA1*, *BRCA2*	DNA repair, oocyte genome stability, ovarian reserve	Human carrier studies, mouse ovarian models	Indicates convergence on genome stability networks rather than isolated loci alone.
Oxidative stress → granulosa apoptosis [59]	ROS–JNK–p53 axis	Granulosa apoptosis, follicular atresia	Granulosa cells/models	Suggests interaction between stress signaling and broader epigenetic remodeling.
Environmental toxicants → methylation changes [60,61]	Steroidogenic genes, imprinted loci, global 5-mC signals	Steroidogenesis, endocrine disruption	Ovarian tissue, granulosa/cumulus cells, exposure models	Supports distributed regulatory remodeling across functionally related loci.

**Table 3:** Epigenetic biomarker categories in reproductive aging, their proposed clinical uses, and current evidence status.

Biomarker Category	Key Loci/Signature	Sample Context	Intended Clinical Use	Evidence Status and Representative References
Follicular development-associated methylation markers [34]	Ovarian function-related loci and methylation profiles	Granulosa cells (DOR cohorts)	Stratify DOR risk, ART response	Cross-sectional human data
Genome stability-associated epigenetic alterations [47]	*ATM*-, *BRCA1*-related loci	Oocytes; aging ovarian tissue	Mechanistic risk layering	Observational evidence
Follicular activation pathway-linked markers [147]	*PTEN*/*FOXO3* regulatory regions	Animal and limited human datasets	Identify low activation threshold	Functional model evidence
Inflammation-related methylation signatures [148]	Inflammation-related signatures	Aged ovarian microenvironment	Context annotation, subtype stratification	Descriptive profiling
Epigenetic age acceleration metrics [40]	DNAm age acceleration	Peripheral blood	Early risk prediction, aging trajectory	Cohort-based studies
Putative ovarian-derived cfDNA methylation markers [120]	Tissue-derived methylation fragments	Plasma	Non-invasive monitoring concept	Conceptual evidence

## Data Availability

No new data were created or analyzed in this study. Data sharing is not applicable.

## Abbreviations

The following abbreviations are used in this manuscript:

CpG	Cytosine–phosphate–guanine dinucleotide
DNMT	DNA methyltransferase
TET	Ten–eleven translocation dioxygenase
ROS	Reactive oxygen species
POI	Premature ovarian insufficiency
DOR	Diminished ovarian reserve
AMH	Anti-Müllerian hormone
FSH	Follicle-stimulating hormone
AFC	Antral follicle count
cfDNA	Cell-free DNA
PI3K	Phosphoinositide 3-kinase
AKT	Protein kinase B
mTOR	Mechanistic target of rapamycin
TGF-β	Transforming growth factor beta
SMAD	Suppressor of mothers against decapentaplegic
